# Improved autobiographical memory with central thalamic deep brain stimulation in traumatic brain injury

**DOI:** 10.1093/braincomms/fcag174

**Published:** 2026-06-05

**Authors:** Eun Young Choi, Brian Levine, Jonathan D Victor, Linda M Gerber, Jaimie M Henderson, Nicholas D Schiff

**Affiliations:** Department of Psychiatry and Behavioral Sciences, Stanford University School of Medicine, Stanford, CA 94305, USA; Department of Neurosurgery, Stanford University School of Medicine, Stanford, CA 94305, USA; Rotman Research Institute, Baycrest Academy for Research and Education, Toronto, ON, Canada M6A 2E1; Department of Psychology, University of Toronto, Toronto, ON, Canada M6A 2E1; Department of Medicine, Division of Neurology, University of Toronto, Toronto, ON, Canada M6A 2E1; Feil Family Brain and Mind Research Institute, Weill Cornell Medicine, NewYork City, NY 10065, USA; Department of Neurology, NewYork Presbyterian Hospital, New York City, NY 10065, USA; Department of Population Health Sciences, Weill Cornell Medicine, NewYork City, NY 10065, USA; Department of Medicine, Weill Cornell Medicine, NewYork City, NY 10065, USA; Department of Neurosurgery, Stanford University School of Medicine, Stanford, CA 94305, USA; Feil Family Brain and Mind Research Institute, Weill Cornell Medicine, NewYork City, NY 10065, USA; Department of Neurology, NewYork Presbyterian Hospital, New York City, NY 10065, USA

**Keywords:** central thalamus, deep brain stimulation, autobiographical memory, traumatic brain injury, central lateral nucleus

## Abstract

Memory impairment is a common, debilitating consequence of moderate-to-severe traumatic brain injury, affecting daily life functions. There are few treatment options for memory impairment, particularly for real-life autobiographical memory. Recently, in a first-in-human Phase I clinical trial, we demonstrated the improvement of executive attention and arousal in five participants with moderate-to-severe traumatic brain injury using deep brain stimulation of the central thalamus, specifically the central lateral nucleus and its projecting fibres within the medial portion of the dorsal tegmental tract [Schiff *et al*. (Thalamic deep brain stimulation in traumatic brain injury: A phase 1, randomized feasibility study. *Nat Med* 2023; 29: 3162–74.)]. Here in this within-subject study, we report a concomitant improvement in autobiographical memory in all five participants. Participants were tested on their ability to recall autobiographical memories using a cue word-based recall task, and on the specificity of memories using the Autobiographical Interview [Levine *et al*. (Aging and autobiographical memory: Dissociating episodic from semantic retrieval. *Psychol Aging* 2002; 17: 677–89.)], before the start of treatment and at sessions ∼3, 6, 9 or 13 months thereafter. Participants also provided self-reports of autobiographical memory changes during treatment. All five participants showed an increase in the average number of autobiographical memories recalled with treatment (range: 12–100%, average: 45.9%, *P* = 0.032). Autobiographical Interview testing of the last four participants showed an average increase with treatment in the specificity of the recalled autobiographical memories, as measured by the per cent of episodic (temporally- and spatially-specific to the memory) details out of the total episodic and semantic (factual, not memory-specific) details (18.7% improvement; main effect of treatment time *F*(3,9) = 5.85, *P* = 0.017). In subjective self-reports, four of the five participants clearly endorsed autobiographical memory improvements in their daily lives, collectively in the detail and vividness of the memories, the frequency of memories recalled, and the recollection of memories from time periods of post-traumatic retrograde amnesia. These results raise the possibility that the central thalamus modulates the autobiographical memory system, potentially via the glutamatergic connections of the targeted fibres emanating from the central lateral nucleus. The increased recall and specificity of autobiographical memories following treatment suggest that central thalamic deep brain stimulation may work to stabilize and refine the multi-faceted process of autobiographical memory retrieval, resulting in improved memory and, in turn, more effective everyday function. These findings support the possible use of central thalamic deep brain stimulation for improving autobiographical memory in moderate-to-severe traumatic brain injury.

## Teaser

Impaired autobiographical memory (AM) (memory of personal events) is a debilitating consequence of traumatic brain injury), affecting daily life. There is no established effective treatment. Choi *et al*. report that central thalamic deep brain stimulation improved AM in five individuals with traumatic brain injury.

## Graphical Abstract

**Graphical Abstract:**
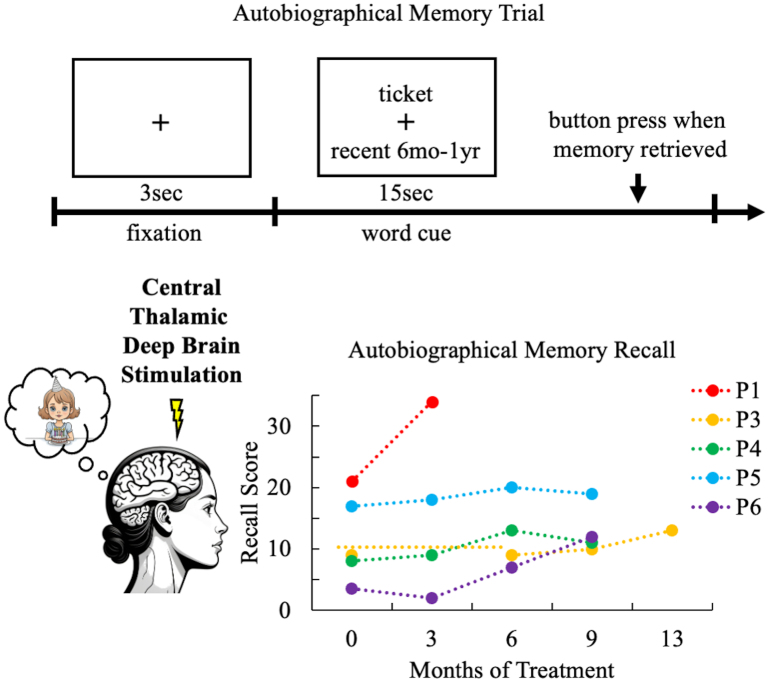

## Introduction

Moderate-to-severe traumatic brain injury [msTBI; Glasgow Coma Scale (GCS) = 3–12, Glasgow Outcome Scale-Extended (GOS-E) = 5–7] results in a broad array of behavioural deficits, including poor memory, poor executive function and fatigue.^1,2^ While most memory research in msTBI concerns memory as assessed by laboratory tasks,^3^ real-life mnemonic tasks necessary for core life functions, such as social interactions and future planning, rely on autobiographical memory (AM; memory for facts and events from one’s life).^4-7^ In TBI, AMs have fewer memory-specific details, reduced temporal scope, and are associated with disruptions to such core life functions.^8-10^ Despite the importance of AM deficits in msTBI, only a few studies have explored treatments, such as behavioural,^11^ pharmacological,^12^ and neurofeedback^13^ interventions.

The multi-faceted nature of AM retrieval is reflected in the dynamic engagement of distributed brain regions underlying mnemonic search, retrieval, monitoring, and narrative coherence.^14^ These regions include the medial prefrontal cortex (mPFC), posterior cingulate cortex (PCC), retrosplenial cortex (Rsp), precuneus and the medial temporal lobe (MTL) memory system, which overlap with regions of the default mode network.^15,16^ In TBI, structural, functional, and metabolic abnormalities have been observed in these regions, including reduced brain volume,^17-19^ reduced neuronal glucose uptake,^20,21^ evidence for partial deafferentation and functional down-regulation underlying executive dysfunction,^22-24^ and abnormalities in functional connectivity.^25,26^

How might AM deficits arise in TBI? A prominent process that occurs following a TBI is the downregulation of the central lateral (CL) nucleus of the thalamus and the broader arousal regulation system.^27,28^ CL is a key node in the arousal regulation system that receives inputs from the brainstem arousal centres, including the reticular nuclei,^29,30^ and is connected with regions throughout the brain, including in the cerebral cortex and striatum,^31^ primarily via AMPA receptor glutamatergic connections.^32^ The mesocircuit hypothesis posits that based on CL’s widespread connections, the diffuse axonal damage following a TBI disproportionately downregulates CL, which in turn downregulates CL’s targets.^33^ Chief among CL’s targets are attention-related regions in the dorsomedial prefrontal cortex and dorsal anterior cingulate cortex. This mechanism is thought to underlie the commonly observed cognitive deficits in TBIs, despite heterogeneity in the primary structural damage; as well as several demonstrations of instrumental improvements in arousal and cognitive function following CL stimulation. Evidence for the critical role of CL includes a macaque study in which CL stimulation rescued fatigue-induced performance deficits in an attentional vigilance task^34^ and two human studies in which CL stimulation restored a minimally conscious participant to a conscious, behaving state^35^ and improved executive attention and arousal in five msTBI participants.^36^

While CL’s attention and motor-related connections are well known, its connections pertaining to memory are less recognized, but nonetheless present. Animal tract-tracing shows that CL is not directly connected to any structures in the MTL memory system.^31,37-41^ However, in macaques, a subset of CL’s projections target cortical midline areas that are homologous to regions of the human default network involved in AM.^42-45^ In macaques, demonstrated targets include areas in the mPFC [Monkey Brodmann Area (BA) 24b], PCC (Monkey BA 23a), Rsp (Monkey BA 30) and precuneus (Monkey BA 31, PGm, PEc, PEci;^43,46,47^ see also rodent study^31^) (Note: tracer studies thus far report that CL receives afferents from, but does not send efferents to, the caudal inferior parietal lobule, another region of the default network^48,49^). In turn, mPFC, PCC and Rsp are connected with the subicular complex,^50-52^ entorhinal cortex,^53-56^ perirhinal cortex (Monkey BA 35, 36) and parahippocampal cortex (Monkey areas TF, TH).^50,55-59^ Thus, downregulation of CL in TBI may lead to the downregulation of mPFC, PCC and Rsp, which in turn downregulate MTL structures, thereby reducing functions related to AM. Indeed, rat studies have shown that deep brain stimulation (DBS) of CL improves object recognition memory,^60^ as well as rescues spatial memory in an amyloid beta-infused rodent model of Alzheimer disease.^61^ In addition, although CL is not directly connected to the hippocampus, these studies, respectively, reported gene expression changes and rescue of glutamatergic spine density in the hippocampus following CL stimulation, indicating CL’s ability to indirectly modulate the hippocampus. However, it is yet unknown whether CL and its downstream glutamatergic pathways modulate a high-order form of memory such as AM.

Here, we tested the hypothesis that CL stimulation remediates AM deficits in five msTBI participants. These participants underwent DBS of the central thalamus, specifically targeting the CL nucleus of the thalamus and its projection fibres within the medial portion of the dorsal tegmental tract (CL/DTTm) in a Phase I clinical trial (CENTURY-S) designed to test its effects on dysexecutive attention and fatigue.^36,62^ Our test battery separately assessed distinct AM processes: AM generation (searching for a memory based on a cue) and AM elaboration (the richness of AM given an identified memory).^14,63^ To assess the generative component, we used a cue word-based AM recall task closely adapted from a well-validated paradigm^64,65^ to test AM recall ability before and after the start of CL/DTTm DBS treatment. To assess the specificity of the elaborative component, we examined the percentage of episodic (spatiotemporally-specific to the memory) out of the total number of episodic and semantic (factual, non-specific to the memory) details quantified using the Autobiographical Interview (AI),^66^ a well-established method for dissociating these canonical categories of AM content that is sensitive to changes in AM in aging and neurodegenerative disease,^67^ MTL damage^68^ and the AM network in numerous structural and functional neuroimaging studies.^69-73^ Among chronic-phase TBI participants administered the AI, reduced specificity (i.e. reduced episodic and increased semantic autobiographical details) was evident in those with severe TBI,^74^ a pattern associated with distributed volume loss in the above-described default mode network regions. On the basis of these findings, we hypothesized that CL/DTTm DBS would address these mnemonic changes by increasing the number and specificity of recalled AMs in narrative AM. These measures were supplemented by self-reports of AM improvements experienced by the participants in their daily lives.

## Materials and methods

### Study design

This study examined the effects of CL/DTTm DBS treatment on AM in the five participants using a within-subject design. Participants were enrolled in a clinical trial examining this treatment on executive attention and arousal.^36^ Participants were tested on AM measures before the start of treatment and at sessions ∼3, 6, 9 or 13 months thereafter while receiving treatment during and after the clinical trial. AM recall task data, comprised of a single measure, were analysed using the Wilcoxon signed-rank test. AI data, comprised of multiple measures, were analysed using a repeated-measures ANOVA and Page’s *L* test.

### Participants

Six participants (P1–P6) with msTBI were enrolled in the NIH Brain Initiative CENTURY-S Phase I clinical trial (NIH BRAIN UH3NS095554; ClinicalTrial.gov NCT02881151) investigating the safety and feasibility of CL/DTTm DBS in restoring executive function.^36^ Briefly, all participants provided informed consent according to protocols approved by Stanford University’s Institutional Review Board in accordance with the Declaration of Helsinki. Participant inclusion and exclusion criteria included an age of 22–60 years, a history of msTBI as assessed by a GCS (a measure of consciousness level after TBI) score of 3–12 within the first 48 h of injury, current GOS-E (a measure of functional outcome after TBI) score of 5–7, being two or more years post-injury, and failure to return to the pre-injury level of vocational or educational function. Participant P2 had her DBS system removed due to safety reasons and did not participate further in the trial, resulting in five participants who completed the study. Table 1 shows the demographic information of the participating participants. All participants demonstrated evidence of diffuse axonal injury with generalized atrophy, asymmetric volume loss, and focal injuries (Supplementary Figs 1–5). Of note, P6 demonstrated marked bifrontal structural lesions, including large left hemispheric volume loss within the medial frontal lobe (Supplementary Fig. 5). See Schiff *et al*.^36^ for further details on eligibility criteria and recruitment.

**Table 1:** Demographic information

Participant	Sex	Age (years)	Education (years)	Time since injury (years)	Pre-surgical GOS-E score
P1	F	39	16	20	5
P3	M	60	13	3	5
P4	M	22	13	5	5
P5	M	30	14	10.5	6
P6	M	28	14	9	6

Eighteen healthy adults (7M, 11F, mean age 43.3 ± 12.1 years) were recruited as comparison participants for a study of AM in depression using the AI.^75^ As part of an unpublished follow-up study, their memory for distinct autobiographical events was serially assessed and scored using the AI, providing an independent estimate of the degree to which AM performance changes over time in healthy adults with no intervention. This study was approved by the Baycrest Institutional Review Board, and all participants provided informed consent.

### Surgery and CL/DTTm DBS treatment

Detailed descriptions of the clinical trial, surgery, and treatment are in Schiff *et al*.^36^ Briefly, participants underwent surgery consisting of two stages: (i) implantation of DBS leads (Medtronic, Minneapolis, MN, USA) into the central thalamus (Supplementary Fig. 6), targeting CL and its connections with the dorsomedial frontal cortex located in the DTTm; and (ii) placement of an implantable neurostimulator (P1: Medtronic Activa PC + S; P3–P5: Activa PC; P6: Percept) connected to the DBS leads by extension wires tunnelled from the scalp down to the chest. After post-operative recovery, therapeutic stimulation settings for each participant were determined based on performance on a neuropsychiatric battery (which did not include a test of AM) and avoidance of side effects. Once the therapeutic stimulation setting was determined (Supplementary Table 1), participants underwent a 90-day treatment period in which stimulation was on for 12 h during the day and off for 12 h at night.

### AM recall task

**Figure 1:**
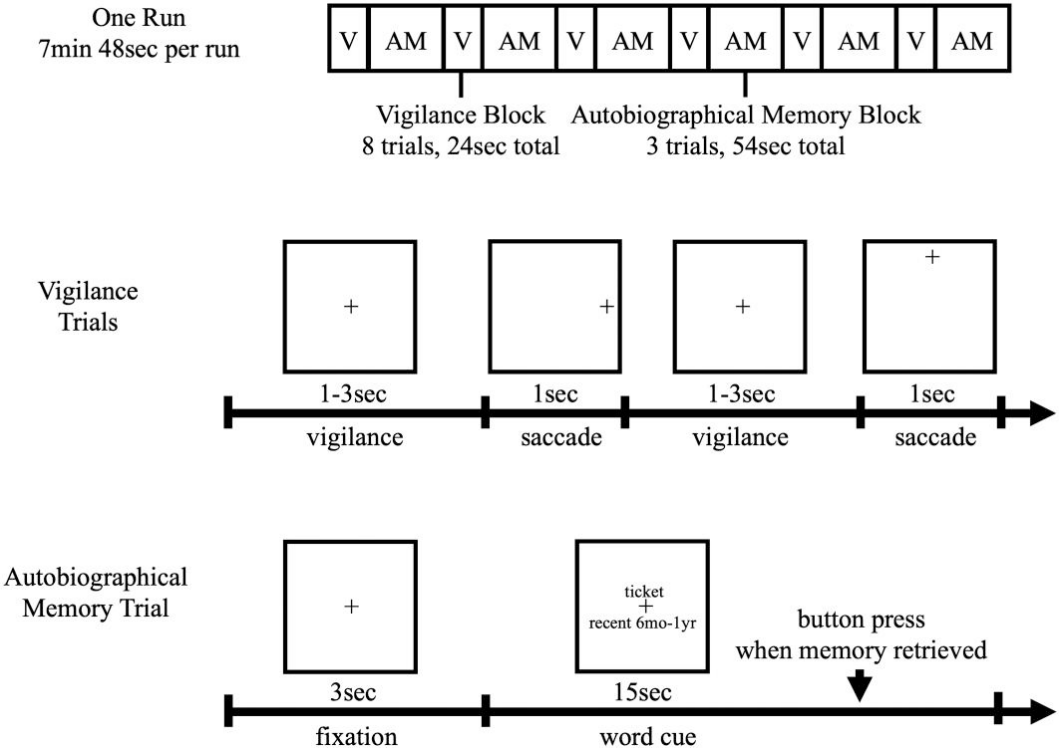
**AM recall task structure.** The task consisted of two runs of AM recall blocks interleaved with Vigilance (V) blocks (not reported here).

The AM recall task was presented combined with a visually-guided attentional vigilance task (‘Vigilance-AM task’; data not reported here). There were two runs, each run consisting of six blocks of eight vigilance trials that were interleaved with six blocks of three AM recall trials (Fig. 1; the interleaved design was originally selected to time-efficiently administer both tasks in the intraoperative setting). A vigilance trial consisted of a variable delay ‘vigilance’ fixation period (1–3 s, mean = 2 s) with a centrally located crosshair, and then a 1-s saccade target presented at the top, bottom, left or right part of the screen at 10° of visual field eccentricity from the centre, to which the participant was required to saccade within the 1-s window. All four saccade targets were randomly presented with equal frequency within one block (six trials per target per block).

An AM recall trial consisted of a 3-s fixation period with a centrally located crosshair, followed by a 15-s period in which the central crosshair remained on the screen, while a cue word appeared immediately above the crosshair and one of two memory time periods (‘distant’ pre-injury and ‘recent’ post-injury within the last 6–12 months; see Supplementary Table 2 for participant-specific distant time periods) appeared immediately below the crosshair. A script of the task instructions was read by the experimenter to the participants prior to testing at each timepoint. Participants were instructed to keep their eyes fixated on the crosshair at all times. Once the cue word and memory time period appeared, they were instructed to maintain fixation while reading the words with their peripheral vision. This was done to minimize muscle and eye movement during the simultaneously recorded EEG for P1 and P6 who were tested in the clinic (data not reported here), and to maintain consistency in the task for P3, P4 and P5 who were tested without EEG via video conferencing due to COVID-19 pandemic restrictions on in-person testing. Participants were instructed to recall an AM that occurred within the indicated time period. The AM did not have to be related to the cue word above the crosshair. Once an AM was remembered, participants pressed a button to indicate successful recall, after which they were instructed to relive the AM in as much detail as possible until the trial ended. Participants were given the opportunity to take a 5-min rest between the two runs, although not all participants rested for the full time. After the completion of both runs, participants took a post-test questionnaire that reviewed each AM trial and asked whether an AM had been recalled; and if so, briefly what its content was, the participant’s alertness during the memory recall, and the valence of the memory. Alertness was measured on a 5-point scale consisting of 1 (‘Not at all’), 2 (‘Slightly’), 3 (‘Moderately’), 4 (‘Very’) and 5 (‘Extremely’). Valence was measured on a 5-point scale consisting of −2 (‘Strongly negative’), −1 (‘Somewhat negative’), 0 (‘Neutral’), +1 (‘Somewhat positive’) and +2 (‘Strongly positive’).

Five versions of the Vigilance-AM task were created for a pre-treatment start timepoint and 3, 6, 9 or 13 months after treatment start timepoints. Each version had a different schedule of saccade targets and variable delay fixation periods, as well as novel sets of 18 cue words. Cue words were culled from the Toronto Word Bank and matched for imagery, concreteness, and frequency in the English language, both across the two memory time periods within a task version and across the four task versions.

Testing occurred prior to the start of treatment, and at 3, 6, 9 or 13 months after the start of treatment. Table 2 shows each participant’s specific timepoints and small deviations in these timepoints for Participants 3 and 5 due to scheduling issues, as well as two baseline timepoints for P6. Table 2 also indicates whether CL/DTTm DBS was on or off at each testing session. In general, participants who were tested in the clinic (P1 and P6) had their CL/DTTm DBS turned off for testing and back on afterwards by the investigators. P3, P4 and P5 had to be tested remotely due to COVID-19 safety restrictions. For these remote testing sessions, CL/DTTm DBS was left on either at the participant’s request to not interrupt treatment or due to concerns that the therapeutic stimulation might not be reliably turned back on by the participant after testing.

**Table 2:** Testing timepoints

Participant	Baseline 1	Baseline 2	3 Months treatment	6 Months treatment	9 Months treatment	13 Months treatment
P1						
Timepoint exceptions						
DBS on/off during testing	OFF		OFF			
AM recall task	X		X			
AI task						
P3						
Timepoint exceptions				5 months a	8 months a	
DBS on/off during testing	OFF			ON	ON	ON
AM recall task	X			X	X	X
AI task	X			X		X
P4						
Timepoint exceptions						
DBS on/off during testing	OFF		ON	ON	OFF	
AM recall task	X		X	X	X	
AI task	X			X	X	
P5						
Timepoint exceptions				7 months^a^	10 months a	
DBS on/off during testing		OFF	ON	ON	ON	
AM recall task		X	X	X	X	
AI task		X	X	X	X	
P6						
Timepoint exceptions						
DBS on/off during testing	OFF	OFF	OFF	ON	OFF	
AM recall task	X	X	X	X	X	
AI task	X	X	X	X	X	

^a^Small deviations in the 6- and 9-month timepoints for P3 and P5.

For in-clinic testing, tasks were presented on a computer screen using LabView. Participants wore a 256-channel high density EEG cap (Electrical Geodesics, Inc.) (data not presented) and observed the screen while resting their heads on a chin rest to reduce head movement. Eye movements were tracked by an EyeLink 1000 eye tracker (data not presented). Millisecond precise task timing was achieved using a photodiode detecting luminance changes on the computer screen corresponding to different task epochs and connected to a National Instruments data acquisition card. Button-press responses were made with a hand-held button responder that was also connected to the data acquisition card. Response times were measured by post-test reconstruction of these events. For testing via video conferencing (Zoom Communications, Inc.), tasks were run on the experimenter’s laptop and presented by screen share; EEG, eye tracking, and millisecond response times were not measured. However, saccades during the vigilance task were observed by the experimenter and button press responses during the AM recall task were recorded instead by key presses in the chat window. All testing sessions in the clinic and over video conferencing were video and audio recorded.

AM recall scores were calculated for each timepoint as the number of trials in which AMs were successfully recalled out of a total of 36 trials. Successful AM recall was defined as the recollection of a personally occurred event that occurred within a specific time and place, was from the cued memory time period, was retrieved within the 15-s time window as indicated by button press, and was confirmed as such in the post-test questionnaire. Recollections were counted as not successful AM recalls if they did not meet these criteria, if the participant provided a vague post-test description that could not confirm a successful AM recall, or if the AM was a repeat of a previously recalled AM within the same testing session. See Supplementary Table 3 for the number of trials with successful AM recall (‘Correct’), no successful AM recall (‘Incorrect’) or nothing recalled (‘Omit’). A significant difference between the baseline and average treatment memory recall scores was assessed across participants with a one-tailed Wilcoxon signed rank test (*signrank* function; Matlab R2020b, The Mathworks, Inc.).^76^ The effect size for the Wilcoxon signed rank test was calculated as *r* = abs(*Z*-statistic)/√(number of participants).^77^ The one-tailed test was selected based on prior human and rodent stimulation work^35,36,60,61^ indicating that CL/DTTm DBS would improve cognitive functions, including memory. The average treatment memory recall score was analysed to at least partially average out confounds related to differing testing timepoints and COVID-related alterations in testing (such as DBS on/off status) that might have affected performance at certain timepoints.

### AI task

Beginning with P3, the AI task^66^ was added to the study procedure. The AI task was administered to P3, P4, P5 and P6 at baseline and at ∼3, 6 and 9 months (Table 2), one week after completing the Vigilance-AM task. Exceptions were P3, who was tested only at baseline at 6 months of treatment, and P4 who was tested only at baseline, 6 months, and 9 months of treatment. CL/DTTm DBS was on or off during testing as indicated in Table 2. For each timepoint, six AMs were selected from the preceding Vigilance-AM task, comprised of three AMs from each of the distant/pre-injury and recent/post-injury time periods, matched as a group by time period for alertness, valence, and importance ratings (Exception: P6 was tested on four, two, and three AMs for the two baseline and 3-month timepoints, respectively, due to insufficient AMs available from the preceding Vigilance-AM task). AMs were also selected to not have been recalled in AI testing from a prior timepoint, not be embarrassing or traumatic, and not potentially reveal personal information that the participant may not want to share. The AI was administered using standardized instructions.^66^ Briefly, for each AM, participants were asked to recall the memory in three stages: a free recall in which the participant recalled the memory with no prompting by the experimenter, a general probe in which the experimenter prompted open-endedly for more details (e.g. ‘Tell me more about…’, ‘Can you remember anything else about …’), and a specific probe in which the experimenter asked specific questions seeking further details (‘Where did this memory take place?’, ‘What colors, smells, or tastes do you recall in the memory?’, ‘Where were you situated in the room?’). Personal health information was removed from the responses, which were then transcribed by a medical transcription company (TranscribeMe; www.transcribeme.com). In order to avoid introducing bias, transcribed text was separated into individual memories, de-identified, and scored in a random order such that the independent scorer, who had not collected the data, was blind to participant identity and test session. AI scoring entails identification of details (grammatical clauses) that are classified as internal (i.e. episodic, pertaining to events or happenings, temporal, spatial, perceptual, or emotion/thought details specific to the event) or external (i.e. semantic information, repetitions, commentary, or details about other events). For simplicity, the internal and external detail categories are hereafter referred to as episodic and semantic. The scorer had established high inter-rater reliability (intra-class correlation coefficients > 0.90) for internal and external details on the AI scoring method. See Levine *et al*.^66^ for additional details of the AI task.

For each participant’s AM, the total numbers of episodic or semantic details across the three stages (free recall, general probe, specific probe) were counted and used to calculate the per cent of episodic details (out of the total episodic and semantic details). These values were averaged across all AMs to obtain the average total numbers of episodic and of semantic details, which were used to compute the average per cent of episodic details out of the total episodic and semantic details at each timepoint for each participant (AMs were pooled across both baseline timepoints for P6). For P3 and P4, who were tested at a subset of the timepoints, the missing data were imputed with the mean of the average total number of episodic or semantic details across timepoints and subjects; this is a conservative approach as it would tend to underestimate significance and effect size. The results obtained with this method were similar to those obtained by imputing the missing data with data only from the subject, as well as by analysing only the timepoints with data from all four subjects. Per cent change from baseline was calculated using the baseline (average baseline for P6) and average treatment values. Data were further analysed with a repeated-measures ANOVA with Time treated as a categorical variable, Time On Treatment as a fixed-effect factor, and Participant as a random-effect factor. Normality of the data was confirmed using the Shapiro–Wilk test of the data at each timepoint, of the difference scores between timepoints, and of the pooled data across all timepoints. The per-timepoint analysis showed that 11 of 12 timepoints were normally distributed, with only the number of semantic details measure at the 3-month timepoint showing non-normality (*P* = 0.005). Most importantly, the difference score analysis, which is most theoretically relevant for repeated-measures ANOVA assumptions, showed normality for all nine difference score comparisons across all three measures. Effect sizes were determined using partial *η*^2^. Results of the repeated-measures ANOVA were confirmed with the non-parametric Page’s *L* test. Significance was determined using critical *L* values for four subjects and four timepoints (for an increasing trend, critical *L* = 114 for *P* < 0.01 and 111 for *P* < 0.05; for a decreasing trend, critical *L* = 89 for *P* < 0.05).^78^

For the 18 healthy participants, the AI was administered at 0, 1.5 and 2.5 months. While these time intervals differ from those in the present study, the methods of cueing and scoring were comparable to the present study, including the selection of different events at each assessment. These data therefore provide a benchmark as to how much interval change is expected in healthy adults’ successive testing of AM recall without intervention. For each measure, the average scores of AMs extracted from the 1 year (range: 6–18 months) and 10 year (range: 5–15 years) time periods were averaged for each participant at each timepoint. Paired *t*-tests were performed on the data from the 0 and 1.5 months timepoints and from the 1.5 and 2.5 months timepoints.

## Results

### AM recall task

**Figure 2:**
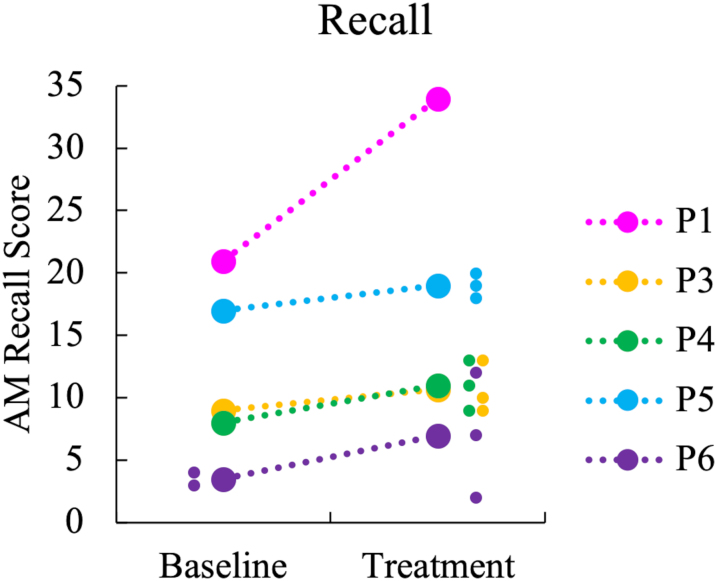
**AM recall performance with CL/DTTm DBS treatment.** For each participant (P), the average baseline and treatment AM recall scores are plotted (large circles), out of 36 possible total points on the AM recall task. The timepoint-specific scores are plotted (small circles) adjacent to the average scores (Note: P1 has single baseline and treatment scores). There was a group improvement of 45.9% (one-tailed Wilcoxon signed-rank *W* = 0, *P* < 0.032) with an effect size of *r* = 0.90. Connecting lines are added to aid in the observation of trends and dashed to indicate that the measures are not continuous.

All participants showed an increase in the average number of memories recalled with CL/DTTm DBS treatment (Fig. 2), with a significant group improvement of 45.9% (one-tailed Wilcoxon signed-rank *W* = 0, *P* < 0.032) with a large effect size (*r* = 0.90) (Table 3; see Supplementary Fig. 7 for a plot of timepoint-specific scores). Individually, there was considerable variation in baseline memory recall score (3–21, out of 36 possible total points) and improvement with CL/DTTm DBS treatment (11.8–100%). P1 and P5 had the highest baseline recall scores (21 and 17, respectively), while P3, P4 and P6 had relatively low scores (9, 8 and 3, respectively). However, baseline recall did not predict improvement with CL/DTTm DBS treatment (Fig. 2). P1 and P6, who had the highest and lowest baseline recall scores, respectively, were the highest improvers (61.9 and 100%, respectively); while P3, P4 and P5, who had intermediate baseline recall scores, were the lower improvers (18.5, 37.5 and 11.8% improvement, respectively). Across participants, there was no significant difference in the memory time period (pre-injury or post-injury) (Supplementary Table 4), or in the ratings for alertness during the recall or valence of the memory, for successfully recalled AMs at baseline versus with treatment (Supplementary Table 5).

**Table 3:** AM recall performance with CL/DTTm DBS treatment

Participant	Baseline	3 Months treatment	6 Months treatment	9 Months treatment	13 Months treatment	Average treatment score	Average treatment—baseline score	Average per cent improve-ment	Average improve-ment effect size (*r*)
P1	21	34				34	13	61.9	
P3	9		9	10	13	10.7	1.7	18.5	
P4	8	9	13	11		11	3	37.5	
P5	17	18	20	19		19	2	11.8	
P6	3.5^a^	2	7	12		7	3.5	100.0	
Average							4.6	45.9	0.90
							*W* = 0, *P* < 0.032		

AM recall scores are out of 36 total points. *P*-value was determined using the one-tailed Wilcoxon signed rank test of baseline and average treatment scores. See Supplementary Fig. 7 for a plot of these data. ^a^ Average score of two baseline timepoints.

### AI task

**Figure 3:**
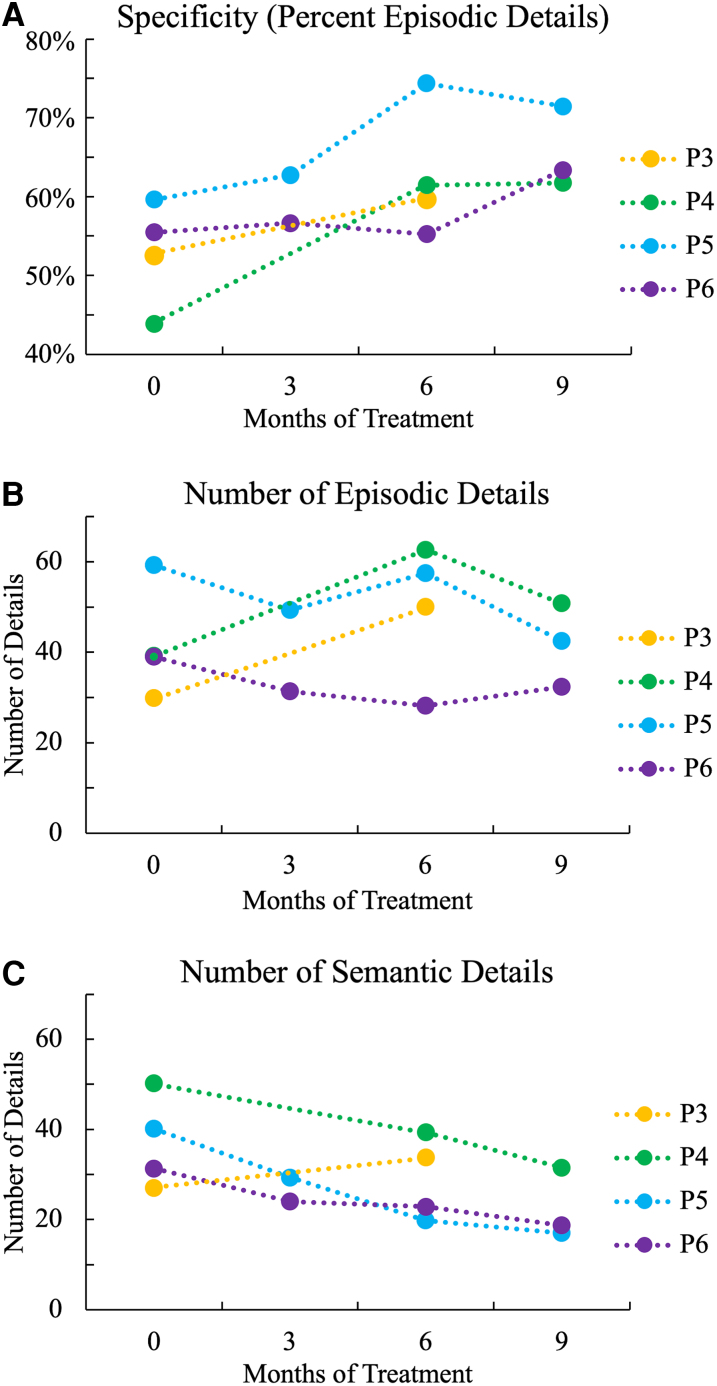
**AI performance with CL/DTTm DBS treatment.** Plots show patient scores at varying treatment timepoints for **(A)** specificity (per cent of episodic details out of the total number of episodic and semantic details), **(B)** the total number of episodic details and **(C)** the total number of semantic details are shown at each timepoint. Connecting lines are added to aid in the observation of trends and dashed to indicate that the measures are not continuous. Following treatment, the specificity increased (average 18.7% improvement, ANOVA: main effect of time *F*(3,9) = 5.85, *P* < 0.017, partial *η*^2^ = 0.66), driven largely by a reduction in the number of semantic details (average 23.4% reduction, ANOVA: main effect of time *F*(3,9) = 3.12, *P* < 0.081, partial *η*^2^ = 0.51), indicating more focused and specific recall of AMs. There was no significant change in the number of episodic details with treatment (average 12.7% improvement, ANOVA: main effect of time *F*(3,9) = 0.83, *P* < 0.51, partial *η*^2^ = 0.22). Note: imputed values at missing timepoints for P3 and P4 (see Table 4) used in the statistical tests are not plotted here. P, Participant.

In order to understand if CL/DTTm DBS treatment affects the specificity of recalled AMs, we measured the percentage of episodic details (out of the total number of episodic and semantic details) for P3, P4, P5 and P6 at baseline and 3, 6 and 9 months of treatment (Fig. 3A; Table 4). All four participants showed an increase in specificity from baseline and across the treatment timepoints with a large overall effect size (average 18.7% improvement, main effect of time *F*(3,9) = 5.85, *P* < 0.017, partial *η*^2^ = 0.66). Examining the underlying numbers of episodic and semantic details (Fig. 3B and C) showed that this increased specificity was driven by a large (but not quite significant) reduction of semantic details over time for P4, P5 and P6 (−33.3, −45.1 and −30.4%, respectively) (average 23.4% reduction, main effect of time *F*(3,9) = 3.12, *P* < 0.081, partial *η*^2^ = 0.51). The change in the number of episodic details was mixed across the participants, increasing for P3 and P4 (54.4 and 33.9%, respectively) and decreasing for P5 and P6 (−16.0 and −21.5%, respectively). This led to a small, non-significant increase in episodic details with treatment (average 12.7% improvement, main effect of time *F*(3,9) = 0.83, *P* < 0.51, partial *η*^2^ = 0.22). These results were confirmed with the non-parametric Page’s *L* test: specificity significantly increases (*L* = 114, *P* < 0.01), the number of semantic details has a trend towards significantly decreasing (*L* = 91), and the number of episodic details does not significantly change (*L* = 101) with treatment. There was no change in the average vividness rating of the AMs recalled with treatment (Supplementary Table 6).

**Table 4:** AI performance with CL/DTTm DBS treatment

Participant	Baseline	3 Months treatment	6 Months treatment	9 Months treatment	Average treatment	Average treatment—baseline	Average per cent improve-ment	*F*-statistic *P*-value	Average improvement effect size (partial *η*^2^)
	Number of episodic details								
P3	29.8	44^a^	50.0	44 ^a^	46.0	16.2	54.4		
P4	39.2	44^a^	62.7	50.8	52.5	13.3	33.9		
P5	59.3	49.3	57.5	42.5	49.8	−9.5	−16.0		
P6	39.0^b^	31.3	28.2	32.3	30.6	−8.4	−21.5		
Average						2.9	12.7	*F*(3,9) = 0.83 *P* < 0.51	0.22
	Number of semantic details								
P3	27.0	29.6^a^	33.8	29.6^a^	31.0	4.0	14.8		
P4	50.2	29.6^a^	39.3	31.5	33.5	−16.7	−33.3		
P5	40.1	29.3	19.8	17.0	22.0	−18.1	−45.1		
P6	31.3^b^	24.0	22.8	18.7	21.8	−9.5	−30.4		
Average						−10.1	−23.4	*F*(3,9) = 3.12 *P* < 0.081	0.51
	Per cent episodic details (specificity)								
P3	52.5	59.8^a^	59.6	59.8^a^	59.7	7.2	13.8		
P4	43.8	59.8^a^	61.4	61.7	61.0	17.1	39.0		
P5	59.6	62.7	74.4	71.4	69.5	9.9	16.6		
P6	55.5^b^	56.6	55.2	63.4	58.4	3.0	5.3		
Average						9.3	18.7	*F*(3,9) = 5.85 *P* < 0.017	0.66

*F* and *P*-values were determined using the repeated measures ANOVA. ^a^The mean value of episodic or semantic details across all participants and all timepoints was used for imputing the data at this missing timepoint. ^b^Average score of two baseline timepoints.

**Figure 4:**
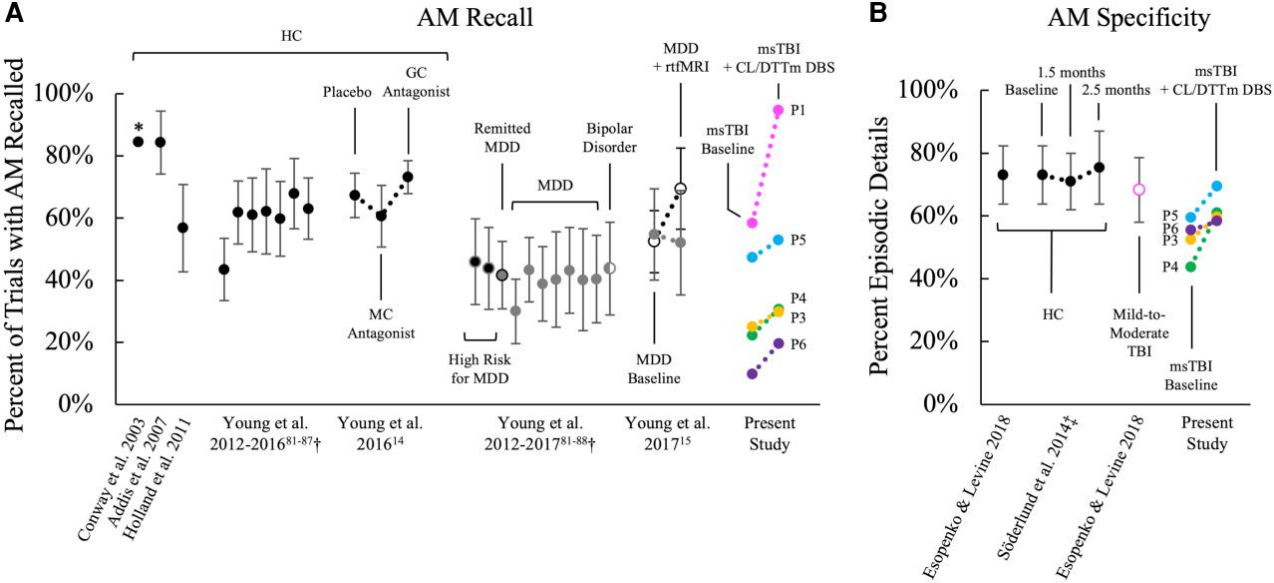
**Comparison of AM recall and specificity improvements with CL/DTTm DBS to the literature.** Baseline and average treatment performances on the **(A)** AM recall task (recall: per cent of all trials with successful recall) and **(B)** AI (specificity: the per cent of episodic details out of the total episodic and semantic details) are compared with group average performances in the literature of patients and healthy controls and under various interventions. AM recall tasks from the literature had the same general structure as the present task, differing in relatively minor ways, such as the number of total trials and the valence of the cue words. The AI also differed in minor ways, such as the number of examined memories. For the healthy control dataset related to Söderlund *et al*.,^75^ only data from the 1 and 10 year time periods were used in order to match the time periods of the present study. Dashed lines indicate repeat testing in the same cohort. Data from the literature show the group average and standard deviation of individual scores. HC, healthy controls; MC, mineralocorticoid; GC, glucocorticoid; MDD, major depressive disorder; rtfMRI, real-time fMRI neurofeedback; msTBI, moderate-to-severe TBI; CL/DTTm DBS, central lateral/medial dorsal tegmental tract deep brain stimulation; P, Participant. *No standard deviation reported. ^†^Each data point in this cluster is from a separate study by Young *et al*.^79-86 ‡^Baseline data were previously published in Söderlund *et al*.^75^

Even though the patients retrieved different events at each timepoint, it is possible that repeated practice with the AI task (and not the intervention) accounted for their improved performance over serial assessments. We therefore analysed AI data from an independent cohort of healthy participants tested at 0, 1.5 and 2.5 months using methods similar to those used in the msTBI cohort. These results showed an average specificity of 72.9 ± 8.2% of episodic details, with 56.6 ± 20.7 episodic details and 22.6 ± 11.7 semantic details, across all timepoints. We found that healthy controls did not systematically improve between timepoints in specificity as assessed by the percentage of episodic details (baseline to 1.5 months: *P* = 0.45; 1.5–2.5 months: *P* = 0.34; Fig. 4B) or in the numbers of episodic and semantic details (baseline to 1.5 months: *P* = 0.10 and 0.70; 1.5–2.5 months: *P* = 0.12 and 0.20, respectively), suggesting that there are no significant practice effects when AM for distinct events is serially assessed in healthy adults without intervention.

## Discussion

In the present study, we report that CL/DTTm DBS led to the improvement of AM in all five participants who completed the CENTURY-S trial, in addition to the increase in executive attention^36^ and decrease in fatigue^62^ reported earlier. There was evidence for CL/DTTm-DBS-related improvement in both generative (search and retrieval) and elaborative (quantity of specific details) elements of AM retrieval. The AM recall task showed an overall 45.9% increase in retrieved memories in response to non-specific cues. The AI task showed that the participants had an average 18.7% increase in specificity with CL/DTTm DBS treatment, potentially by reducing non-specific, semantic detail. These results suggest that CL/DTTm DBS treatment induces greater fluency, focus, and richness of AMs, which are known to be affected in msTBI (as well as other conditions) and with downstream effects on everyday function. Qualitatively, participants and their families reported AM improvements to varying degrees in the fluency of recall, vividness and detail, and accessibility of AMs from periods of post-traumatic retrograde amnesia (Box 1). Remarkably, P1 reported a return to pre-injury levels of AM functioning after 3 months of treatment, as well as several weeks of highly vivid, multi-sensory AM recollections at home during a period of post-trial adjustment of stimulation settings, which resolved when the stimulation voltage was lowered (Box 1). These self-reports are consistent with qualitative improvements broadly in memory reported by all participants and their families in a structured, prospective narrative study accompanying the Phase I trial.^62,87^

Box 1 Participant self-reports. Participants, and their families when available, were asked for their subjective observations of any changes in AM in their daily lives over the course of CL thalamic nucleus/medial portion of the dorsal tegmental tract (CL/DTTm) deep brain stimulation (DBS) treatment. All participants and their families endorsed an improvement in AM at various times during treatment; no participant reported a decrement in AM at any time. P1 reported the greatest improvement, reporting that at 4 months of treatment, there was an ‘explosion’ in her ability to remember ‘highly detailed, very vivid’ AMs, particularly from a pre-injury period that was previously ‘blank’. P1’s mother stated that ‘she started remembering all this stuff … once her mind found the way. It’s happening all the time on a regular basis. It’s just becoming normal, like we’re normal with our memories. … I think [she is] remembering pretty much everything now’. P1 stated, ‘I remember everything about it. … It’s not like I just know a little bit … [it’s] like a picture’. At around 1 year of treatment, P1 additionally reported a 1–2 week period with intense, movie-like AM recollections. These recollections included remembering her grandfather’s wake, such as the green colour of the carpet and the smell of incense in the air, and remembering all the faces and names of the children in her first grade class.

At 3 and 5 months of treatment, P3 did not endorse a strong improvement in AM. However, he reported that his dreams, occurring almost every night, were more vivid and frequent, and included childhood events that he had forgotten about. At 10 months of treatment, P3 reported continuing to have vivid dreams almost every night and added that his recalled AMs were more vivid, as well.

At 3 months of treatment, P4 reported, ‘I believe [AMs have] improved a bit … [there is] a bit more variety of memories from back then I'm recalling…’. However, he still could not remember well the order in which autobiographical events had happened within a constrained time period (e.g. Thanksgiving season).

At 2 months of treatment, P5 reported that he had recalled a few AMs from the 6 months prior to his injury, which was a period of post-traumatic amnesia. At 5 months of treatment, P5 reported that his AMs were ‘brighter’ and more visually detailed, and that he could more thoroughly assess the AM (‘look at it from different angles’), but not necessarily recall more AMs than prior to treatment. At 7 months of treatment, P5 reported having some dreams, which was more frequent than prior to the treatment when dreams were ‘few and far between and can’t [be remembered] one hour after being awake’.

P6 did not strongly endorse an improvement in AMs with treatment. However, at 3 months of treatment, his mother reported an instance of his recalling where he was living and with whom during college, which had surprised her as something he could not have done prior to the treatment.

Studies from the literature provide context to the present results (Fig. 4) using similar versions of the AM recall task and AI to those used here. For the AM recall task, healthy participants performed at the highest range (∼40–84 ± ∼10–14%),^64,65,79-85,88^ followed by participants with depression-associated conditions and bipolar disorder (∼30–50 ± ∼10–16%);^79-86^ as seen in the figure, our participants are at the lowest range (∼10–60% range). Interestingly, two studies by Young *et al*.^12^ examined the effects of potential interventions on recall (specifically, the effects of modulating cortisol levels with various cortisol receptor antagonists and of neurofeedback-based real-time fMRI modulation of amygdala activity^13^); the manipulations modulated recall by ∼7–17 percentage points, which is on the same scale as the improvement in our participants (∼5–36 percentage points) with CL/DTTm DBS. For the AI, healthy controls and mild-to-moderate TBI participants performed similarly at ∼70 ± 10%.^74,75^ Our participants started in the lower range of 44–60% episodic details, consistent with their having cognitive disabilities in the moderate-to-severe TBI range, and had treatment-related improvements ranging ∼60–70%. Altogether, our participants initially performed overall worse than healthy and depression-associated populations, and improved either to levels similar to those of healthy participants or by amounts that are similar to or greater than those observed by other interventions.

Given that different events were selected at each timepoint, there is no reason to expect that memory performance would improve without intervention. Accordingly, no significant practice effects were observed on the AM recall task in 19 major depressive disorder (MDD) participants who were tested twice at an interval of 5–7 days,^13^ as well as on the AI of 18 healthy participants tested at intervals of 1 and 1.5 months (Fig. 4). This lack of practice effects suggests that the improvements we observed are likely due to CL/DTTm DBS, and that the degree of improvement eclipsed the expected variability due to the differing contents of the AMs tested at different timepoints.

We do not believe that the AM improvements are merely due to improved verbal or language function. This is because the participants show an increase in the per cent of episodic details, which corrects for overall verbal output, due to an underlying decrease in semantic details, rather than an increase in both semantic and episodic details, as would be the case if the treatment were simply improving verbal or language function. In addition, no participants had any expressive language impairments at any timepoint in the trial, nor did any endorse verbal or language deficits.

There are several anatomical pathways through which CL/DTTm DBS effects on AM could occur. As CL does not directly connect to the hippocampus and adjacent medial temporal areas,^31,37-41^ the CL/DTTm DBS enhancement of AM recall likely occurs via CL’s connections with cortical regions that process AM, including mPFC, PCC, Rsp and precuneus of the default network. Indeed, CL stimulation in rats was associated with improvements in unrewarded object recognition and spatial memory, and induced changes in gene expression and synaptic density in the hippocampus, indicating the ability of CL stimulation to create long-term changes to this central node of the memory system, despite the lack of direct connections.^60,61^ Consistent with this, CL stimulation in rats lead to an increase in functional MRI blood oxygenation level-dependent (BOLD) activity in the hippocampus and enhanced CL-hippocampal functional connectivity as measured by resting-state functional connectivity MRI; these effects were also seen for regions of direct connectivity, such as the anterior cingulate cortex, somatomotor cortex and Rsp.^89^ However, we note that Liu *et al*.^90^ did not find hippocampal changes in BOLD activity following optogenetic stimulation of CL in rats, although they also observed BOLD activity changes in the anterior cingulate, somatomotor cortex and Rsp; this difference from the Li *et al*.^89^ study could be due to differences in the anaesthesia used or the extent of stimulation from the differing stimulation methods.

The AM tasks used in this study entail complex cognitive operations requiring integrated activity across distributed brain regions, including the mPFC (initiation, arousal, and self-related processes), posteromedial regions (multimodal perceptual-mnemonic integration), and distributed cortical regions involved in the reconstruction of the sensory aspects of mnemonic experiences.^14^ One of the consequences of CL downregulation following TBI may be disruptions to the synchrony in neural activity necessary amongst these regions for successful AM retrieval.^91-93^ Consequently, CL/DTTm DBS may concurrently drive these regions, promoting synchrony amongst these regions and, in turn, driving hippocampal processing and the distributed brain-wide synchrony needed for successful AM retrieval.^68,94^ Accordingly, the present treatment effects showed an increase in the episodic details of AM, which are mediated by hippocampal-neocortical connectivity,^91,93^ whereas semantic details reflecting generic or off-task content did not increase with treatment. Indeed, the significant decline in these semantic details reflects an increased fidelity of recall in response to the task instructions to recall a specific event. It is the episodic elements of AM that are most strongly connected to the representation of the self across time,^95^ which in turn is crucial for self-regulation in everyday situations.^63,96^

It is notable that AMs are predominantly visual^97^ and that CL has several established roles in visual processing (see reviews^98,99^). While we did not observe any changes with treatment in the vividness ratings of the AI (Supplementary Table 6), several participants self-reported changes in the vividness of AMs and dreams in their daily lives (Box 1). CL neurons code eye movement information^100^ and have monosynaptic projections to visual cortex.^101^ Modulation of CL has been demonstrated to alter both the contrast sensitivity of V1 neurons (when electrically stimulated^102^) and orientation tuning when lesioned^103^ or electrically stimulated at low frequencies.^99,104^ Finally, as noted above, CL projects to the precuneus,^47^ which is involved in visuo-spatial imagery and attention^105^ and is a modulatory node of AM recall.^93^ We also note that alongside P3’s report of enhanced vividness in AMs and dreams, functional improvement in P3’s vision emerged with continued treatment after the trial. Prior to the trial, P3 had a large left posterior lesion within the primary visual cortex (Supplementary Fig. 2), along with hemianopia and visual disability requiring a white cane for walking. Within 18 months of CL/DTTm DBS treatment, P3 stopped his use of the walking cane and reported improvements on the Goldmann visual field test in comparison to his pre-surgical baseline score. Taken together, these observations suggest CL/DTTm DBS may enhance visual processing via one or more of these mechanisms, leading to improvements in visual aspects of AM. Further study is needed to specifically examine this hypothesis.

The individual improvements in AM seen with CL/DTTm DBS did not closely parallel the individual improvements in executive function [primary outcome measure: Trail Making Test-B (TMT-B)] in Schiff *et al*.,^36^ consistent with a prior report of no significant correlation between TMT-B and AM.^74^ This variability likely reflects individual differences in the complex underlying connectivity and heterogeneity in TBI-related damage. It also suggests that even though executive attention is a component of AM retrieval, there is some degree of independence between the neural processing underlying AM and executive attention, at least as measured here. Despite this individual variability, we nonetheless found statistically significant group-level AM improvements. A fuller understanding of individual response variability and its implications will require a larger sample size.

The therapeutic potential of the present findings is considerable. Currently, although AM deficits are a major complaint in msTBI and reduce core life functions,^8-10^ there are no established effective treatments for AM deficits in msTBI. The majority of prior studies on the memory effects of DBS have targeted the MTL and associated memory circuitry or the cholinergic system via the nucleus basalis of Meynert using laboratory-based episodic memory, such as verbal memory of word lists (see^106^ for review), in Alzheimer disease and epilepsy. To our knowledge, the present study is the first to quantify improvements in AM with DBS, here in msTBI via the CL-mediated glutamatergic system. Interestingly, P1’s spontaneous at-home episodes of multi-sensory, movie-like re-experiencing of AMs (Box 1) is similar to reports from a fornix DBS study of 42 Alzheimer disease participants, where 20 participants reported spontaneous vivid AM recollections.^107,108^ Of these 20 participants, 6 had multi-sensory recollections involving imagery, smells, and ambient temperature experiences, similar to P1’s experiences. Our finding that stimulating the arousal system at the CL node leads to improvements in AM, as well as arousal and attention-related functions,^36^ indicates that CL/DTTm DBS improves a wide array of cognitive deficits experienced in msTBI. This may differentiate CL/DTTm DBS as a memory treatment from DBS approaches that directly target the memory system.

These data have implications for trial design, specifically the importance of testing at longer timepoints, potentially up to a year. For example, had we stopped data collection after 3 months of treatment, we would not have observed the improvements in AM at later timepoints, and may have concluded that there was no improvement in AM with treatment. In addition, there is evidence that CL/DTTm DBS treatment effects accumulate over the long-term.^35,109,110^ Thus, we suggest that trial designs could benefit from extending over a longer time period to fully capture treatment benefits.

While the present findings provide striking evidence that CL/DTTm DBS improves AM, we note the following caveats. There are only five participants and a number of factors underlying individual variability amongst these participants, including the nature and degree of injury, duration of retrograde amnesia, demographic and premorbid factors such as age, education, and pre-injury cognitive abilities, and day-to-day fluctuations in function due to varying fatigue, mood, and environmental stressors. In addition, due to COVID-related restrictions on being able to turn DBS on and off in the participants who were tested remotely, we can only speculate as to the acute effect of DBS on and off versus chronic effects. Given the known phenomenon of transient symptom improvement due to implant-related microlesion effects in DBS for disorders such as essential tremor and Parkinson’s disease, it is worth considering whether these effects could play a similar role in this study. However, microlesion effects generally persist for only a few days after electrode implantation,^111^ whereas AM improvements in this study occurred many months after implantation—well outside the window of potential transient postoperative symptom improvement. Altogether, these factors necessitate caution when making generalizations from these findings. Further study is needed to understand the impact of these factors with a larger sample size, healthy control data at the same timepoints, and a measure of clinical significance corresponding to improvements in the AM recall task and AI (e.g. using a Reliable Change Index).

We note that there was no confirmation of the veracity of the recall, which could lead to an apparently successful recall of a memory when in fact the memory was recalled in a faulty manner. We followed Addis *et al*.’s^65^ standard protocol of ascertaining twice whether they recalled an AM—the first report is the button press during each trial after a successful AM recall and the second report is the *post hoc* questionnaire asking for details of what they recalled in order to verify that an AM meeting our criteria was recalled.

We also note that DBS delivers a relatively large volume of stimulation, and that structures, other than those explicitly targeted, may be affected. Here, the CL/DTTm axons projecting forward to frontal lobe structures were targeted, as noted in Schiff *et al*.:^36^ the primary, executive attention-related effects of CL/DTTm stimulation likely depend on activation of large, myelinated axons within CL/DTTm, perhaps with additional contribution from axons from the adjacent paralaminar portion of the mediodorsal (MD) thalamic nucleus, which has similar characteristics to CL.^32^ In contrast to paralaminar MD, the medial magnocellular portion of MD is implicated in episodic memory,^32^ and bilateral lesions to this structure produces a profound paramnesia (‘thalamic chronotaraxis’) that alters the memory of time and personal identity.^112^ Nonetheless, we propose that the present findings are less likely to be due to driving magnocellular MD due to its distance from CL/DTTm. In addition, our modelling of the DBS activation of each participant’s connectivity indicated that CL/DTTm DBS maximized activation of CL/DTTm fibres and minimized activation of the adjacent fibres from MD (including both magnocellular and paralaminar MD), centromedian (CM), and ventral posterior lateral (VPL) thalamic nuclei in P1, P3, P4 and P6 participants, and avoided MD fibre activation entirely in P5.^36^ Nonetheless, we cannot eliminate the possibility that the observed AM improvements were due to the stimulation of MD and its memory-related connections.

Finally, based on CL’s neuroanatomical connections to cortical default network regions, we have focused on the effects of CL/DTTm DBS on AM, in addition to executive attention in our prior work.^36^ However, CL/DTTm DBS may improve cognition more generally, including improvements to episodic memory more broadly. Given limited testing time, we prioritized AM over traditional episodic memory tests due to its greater ecological validity and potential clinical significance. We also reasoned that CL/DTTm DBS would be better suited for improving AM, which requires activity across these distributed areas, as opposed to the more limited areas involved in traditional episodic memory tasks.^14^ However, future study is needed to understand the full extent of CL/DTTm DBS on episodic memory and cognition more broadly.

## Conclusion

CL/DTTm DBS treatment improved the recall and specificity of AMs in five msTBI participants, causally demonstrating a new mechanism of interaction between the arousal and AM systems that likely involves long-range cortico-cortical systems modulated by thalamocortical glutamatergic neurotransmission. CL/DTTm DBS may be a promising new treatment for improving both AM and executive attention deficits in msTBI. Future work is needed with additional participants to understand the factors mediating these responses and the potential effect of CL/DTTm DBS on cognition more broadly.

## Supplementary Material

fcag174_Supplementary_Data

## Data Availability

The data underlying this article are available in the article and its online Supplementary material, and upon reasonable request. All analyses were conducted with MATLAB R2020b (www.mathworks.com).
