# Supplementary material for: Improved autobiographical memory with central thalamic deep brain stimulation in traumatic brain injury
Source: Brain Commun. 2026 Jun 5;8(3):fcag174. doi: 10.1093/braincomms/fcag174 (PMC13291816; doi:10.1093/braincomms/fcag174)
Supplement: fcag174_Supplementary_Data [file fcag174_Supplementary_Data.docx]

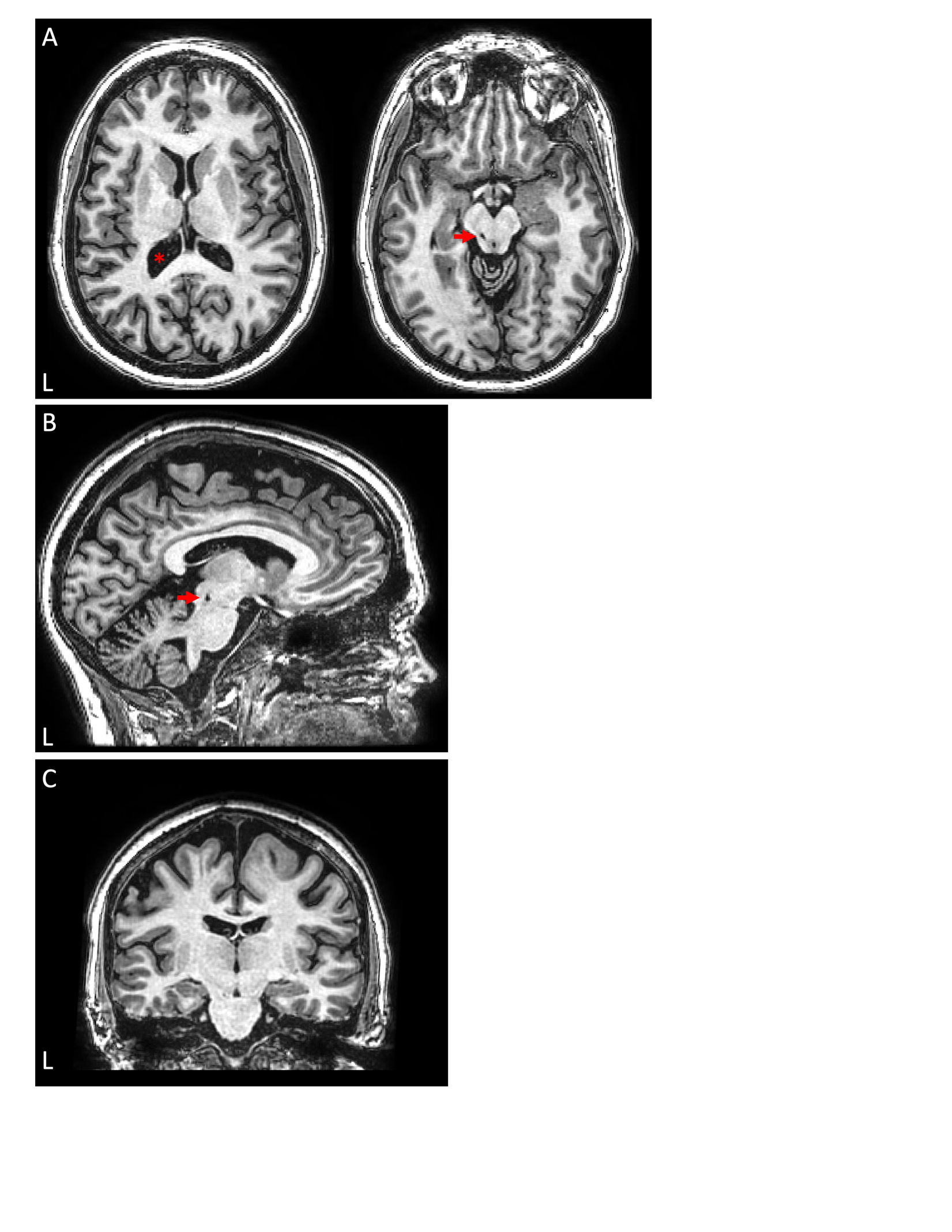


**Supplementary Figure 1. P1 structural brain MRI.** Representative (A) axial, (B) sagittal, and (C) coronal images from P1. Images illustrate increased left hemisphere atrophy near the temporal horn (asterisk) and residua from a small hemorrhage from diffuse axonal injury in the left midbrain (arrows). Coronal image illustrates generalized atrophy. L = Left.


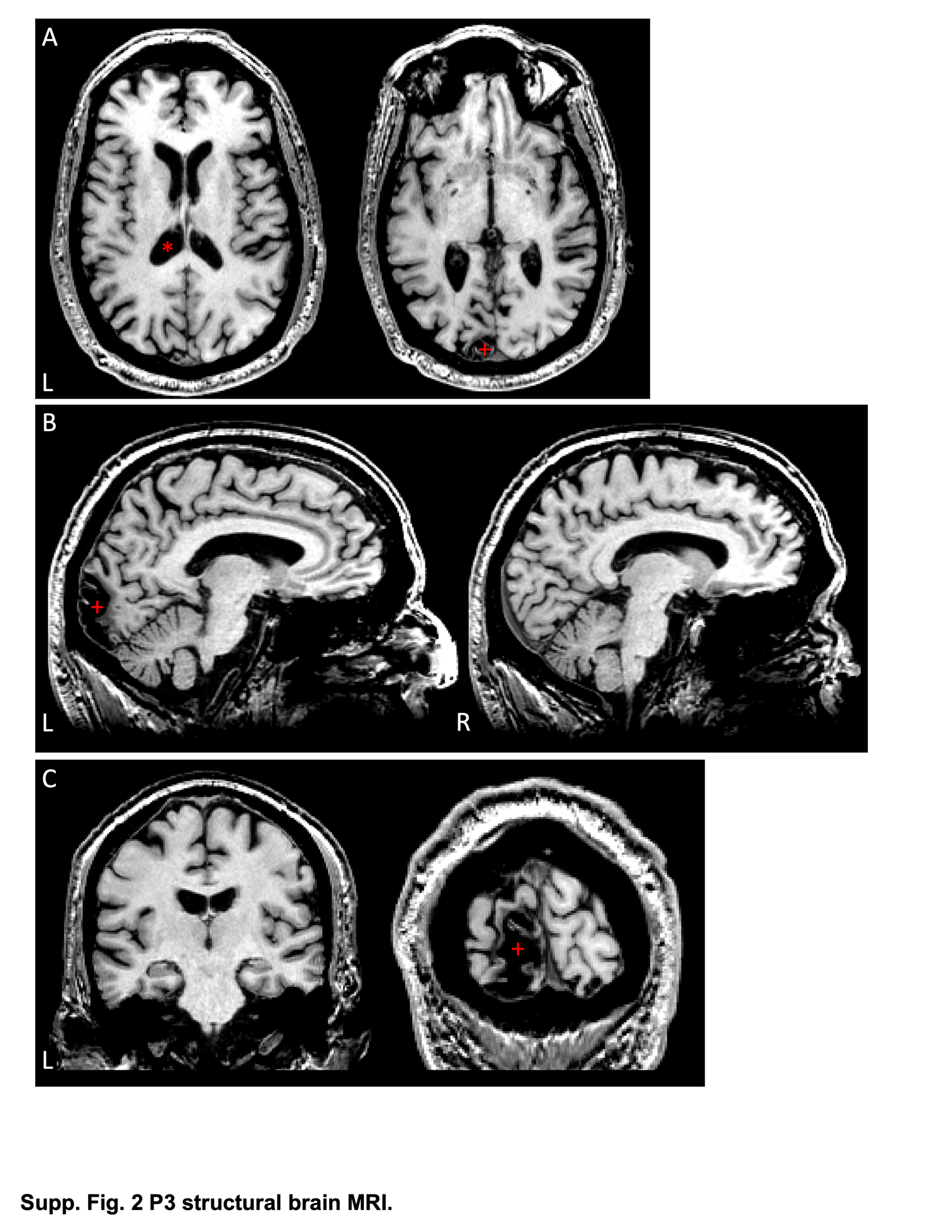


**Supplementary Figure 2. P3 structural brain MRI.** Representative (A) axial, (B) sagittal, and (C) coronal images from P3. Images illustrate increased left hemisphere atrophy near the temporal horn (asterisk) and a left visual cortical infarct (plus symbols). L = Left. R = Right.


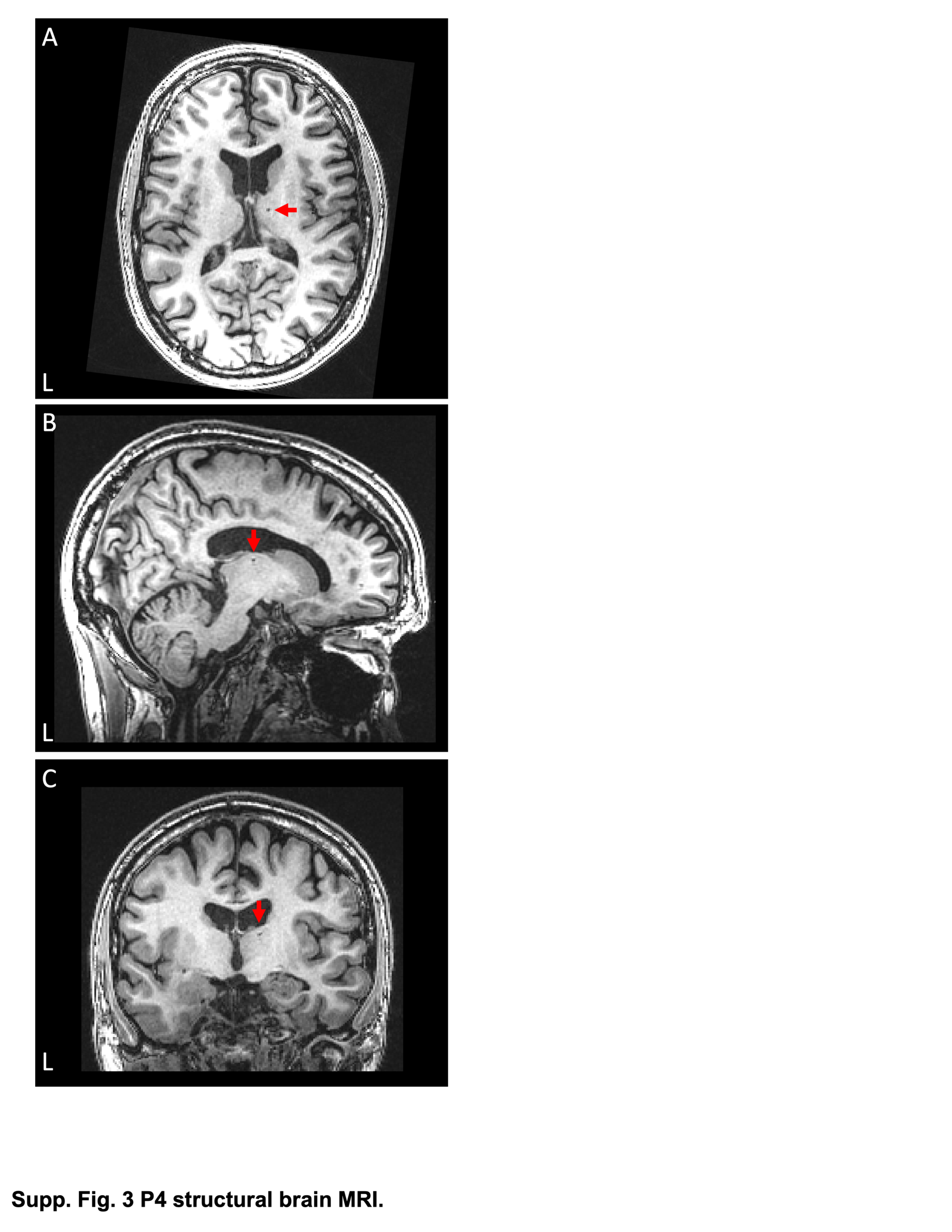


**Supplementary Figure 3. P4 structural brain MRI.** Representative (A) axial, (B) sagittal, and (C) coronal images from P4. Images illustrate right thalamic residua from a hemorrhage secondary to diffuse axonal injury (arrows). L = Left.


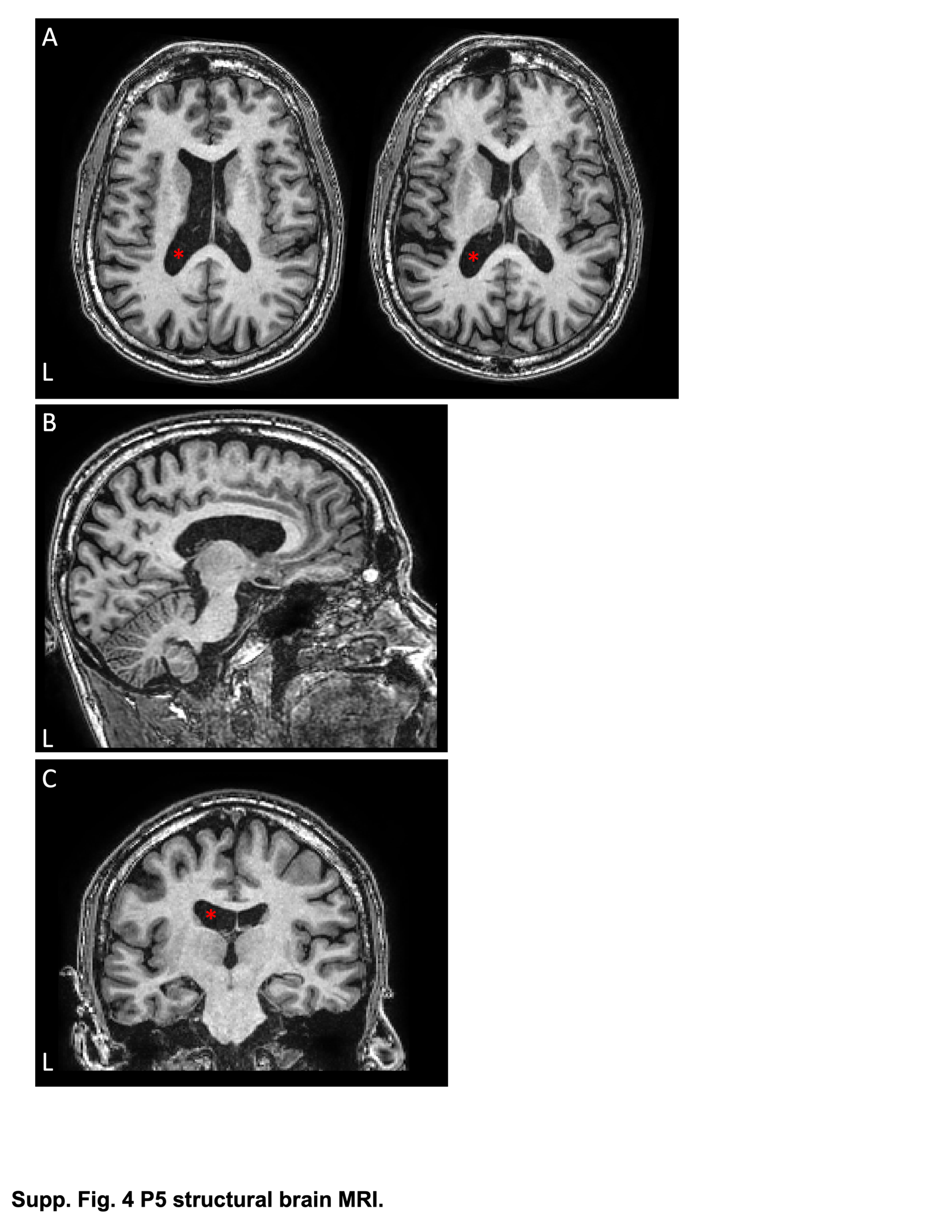


**Supplementary Figure 4. P5 structural brain MRI.** Representative (A) axial, (B) sagittal, and (C) coronal images from P5. Images illustrate dilated left ventricular system marking asymmetric hemispheric atrophy (asterisks). L = Left.


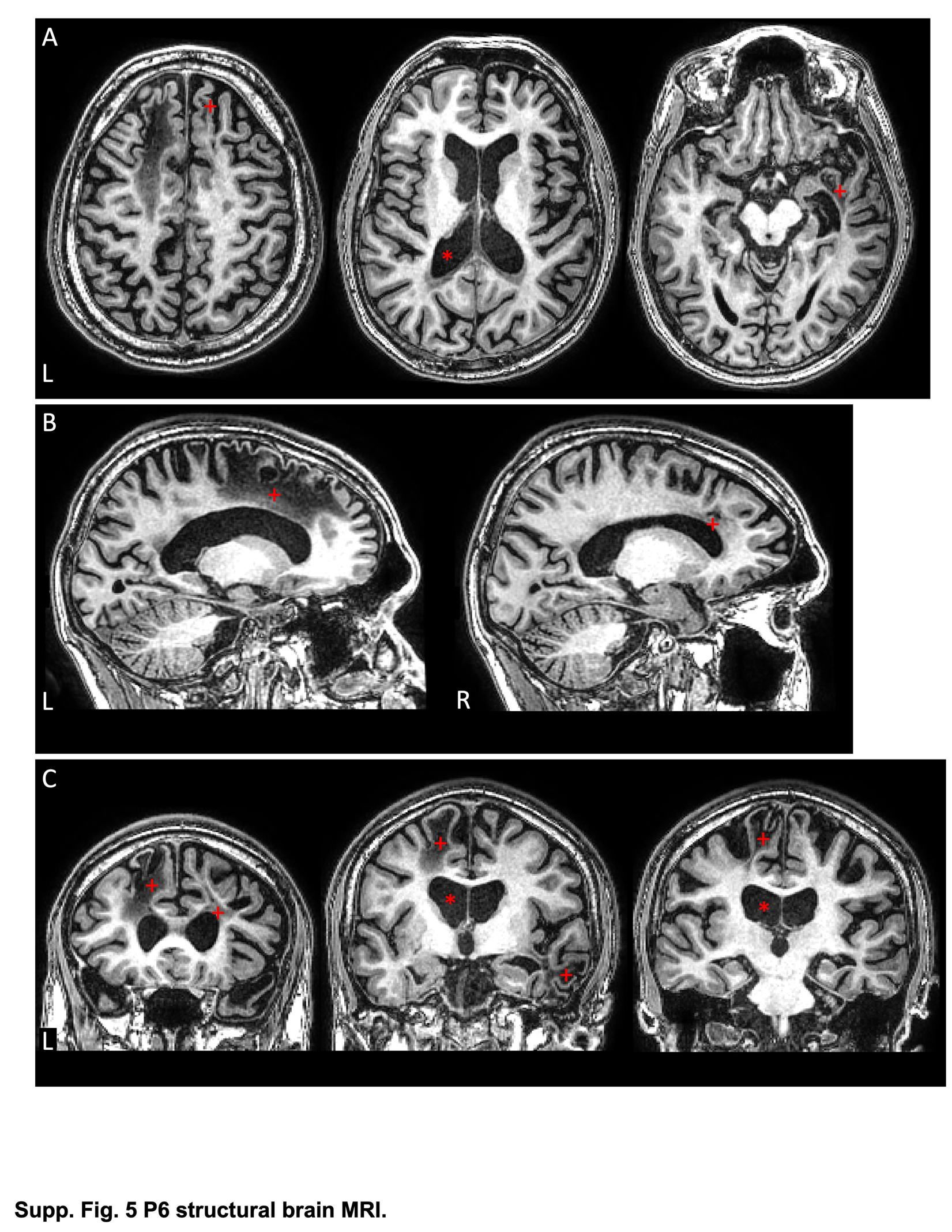


**Supplementary Figure 5. P6 structural brain MRI.** Representative (A) axial, (B) sagittal, and (C) coronal images from P6. Images illustrate severe bilateral fronto-temporal injuries (plus symbols) and left-greater-than-right dilation of the lateral ventricles (asterisks) secondary to the large medial frontal lesion. L = Left. R = Right.

A


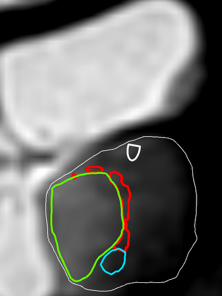

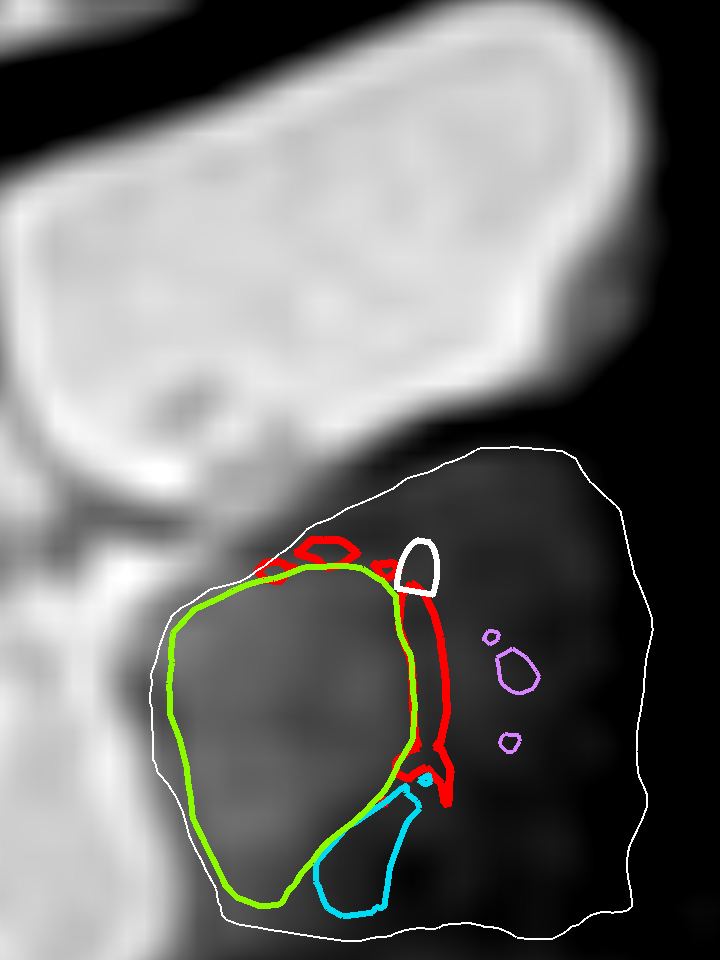


**L3**


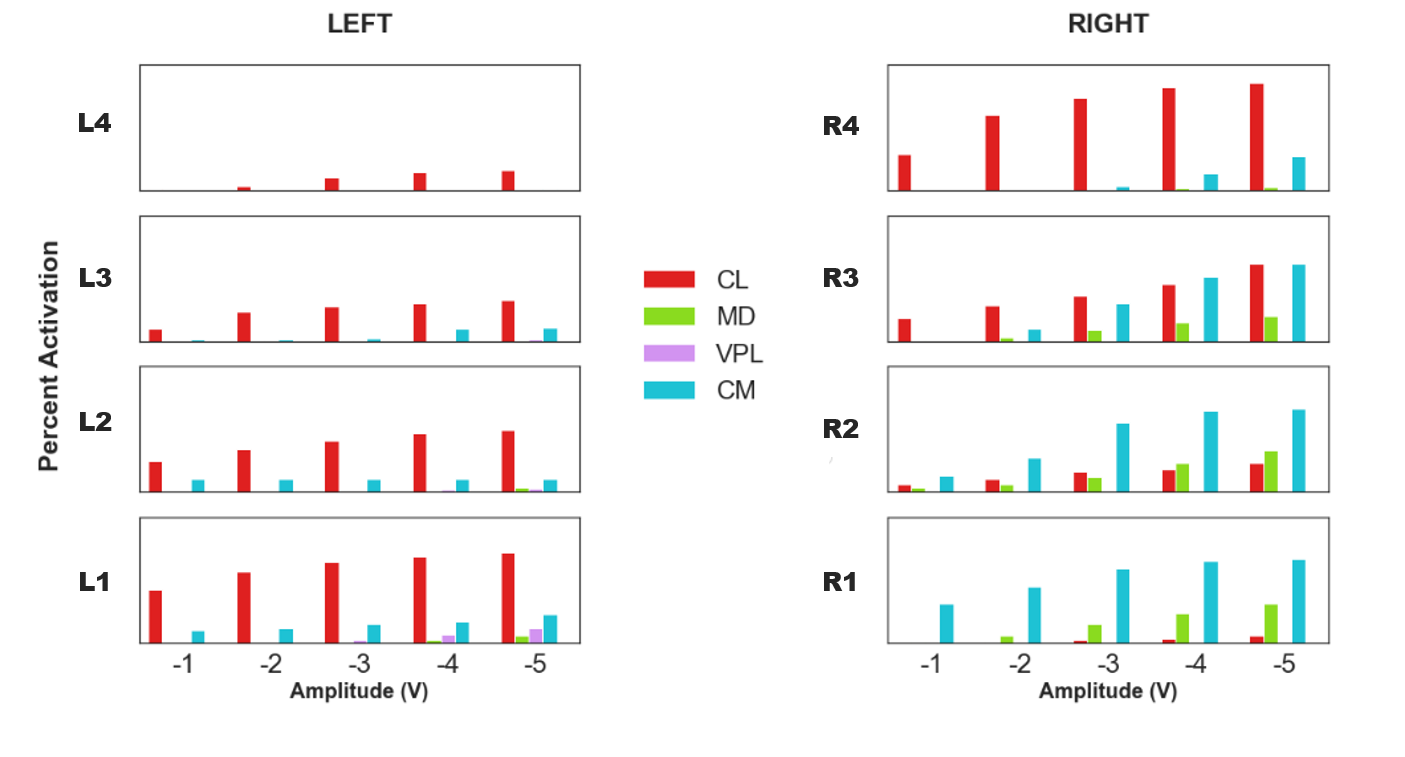


**L4**


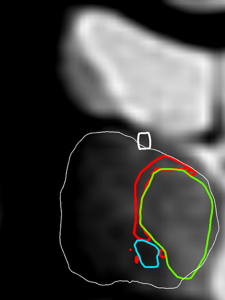

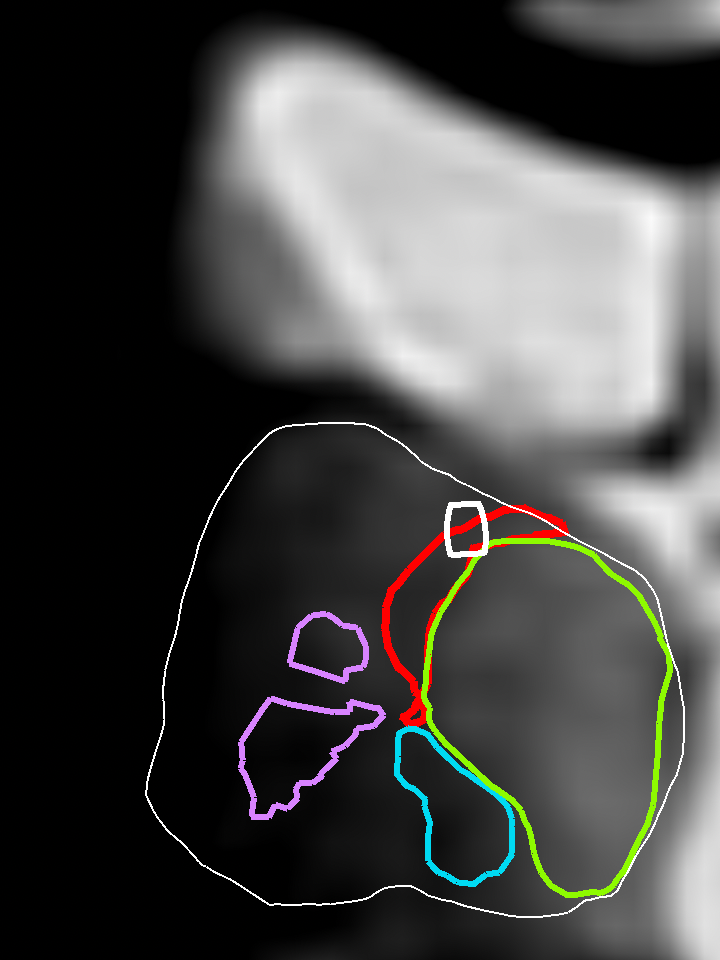


**R4**

**R3**

**
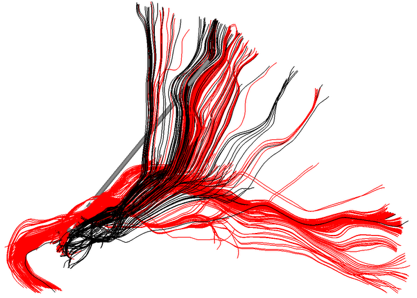
**

B

**
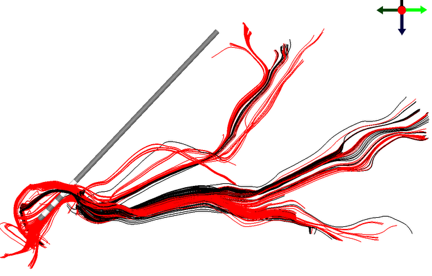
**

**LEFT SIDE**

Front of

Brain

**RIGHT SIDE**

Front of

Brain

DBS

Lead

DBS

Lead

Red: Activated

Black: Not Activated

Red: Activated

Black: Not Activated

**
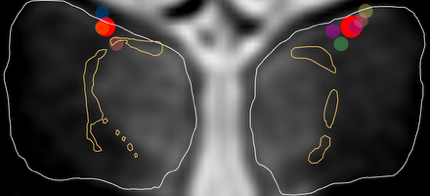
**

C

D

**L4**

**R4**

**P1 P3 P4 P5 P6**

Percent Fiber Activation


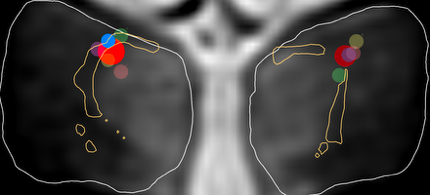

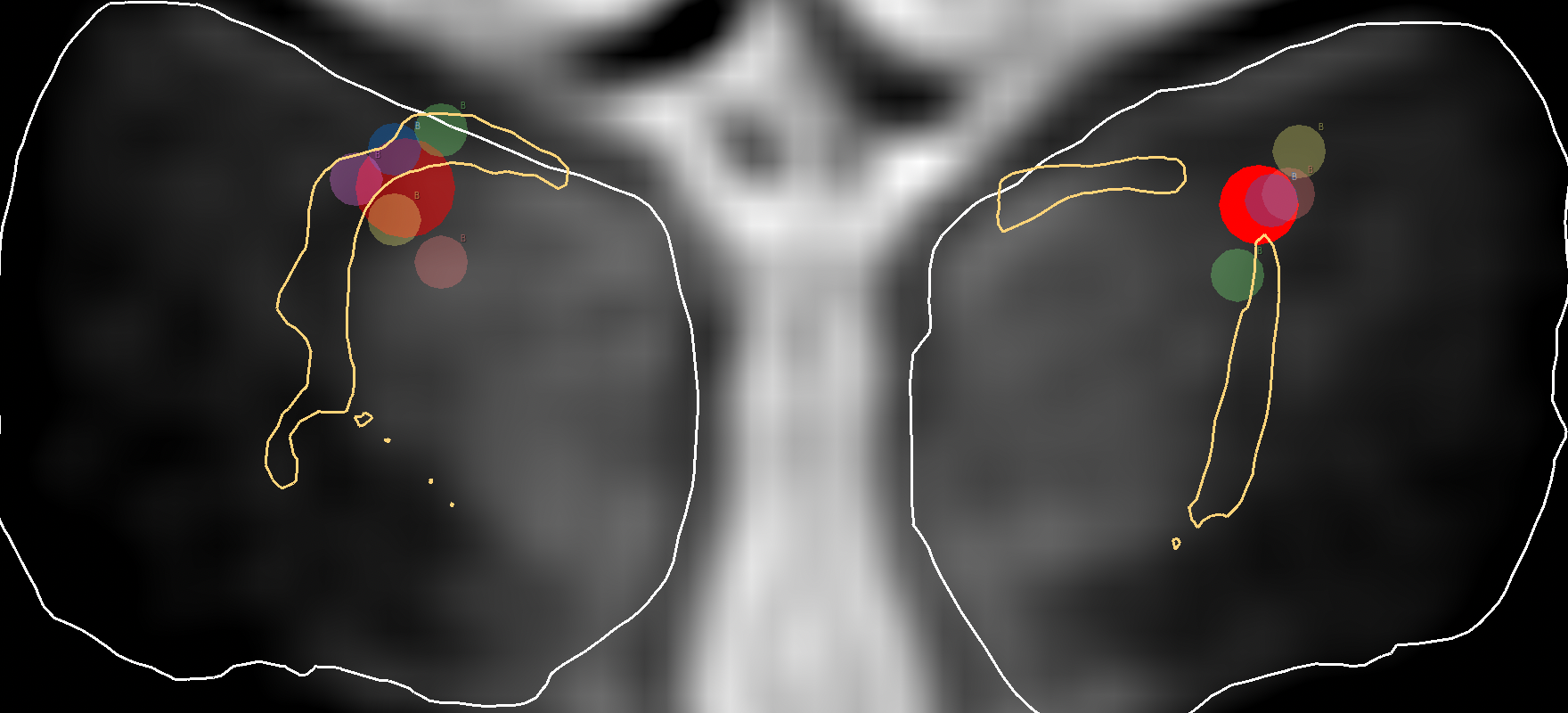


**L3**

**R3**

Centroid of contacts

P1

P3

P4

P5

P6

**Supplementary Figure 6. Activation of CL/DTTm fibers in a representative participant and group summary.** Example placements of the therapeutic DBS contacts (left: L3, L4; right: R3, R4) are shown for participant P4 in **(A)** coronal slices in the participant’s brain and in **(B)** P4’s diffusion tensor imaging model showing that CL’s connections to the prefrontal cortex via the DTTm were activated by DBS. **(C)** Therapeutic contacts for all participants are shown in coronal slices in a study-specific group brain. Light brown outline indicates CL. Small circles show the therapeutic contacts, color-coded for each participant, and rendered dim if out-of-plane or bright if in-plane. Bright red larger circles indicate the group centroid of all top or bottom contacts. **(D)** Histogram showing the percentage of activated fibers that were part of the targeted CL/DTTm fiber tract versus those of adjacent nuclei (MD, VPL, and CM) from each participant’s diffusion tensor imaging model. Note that CL/DTTm was primarily activated over adjacent fibers in each participant. Modified with permission from Schiff ND, Giacino JT, Butson CR*, et al*. Thalamic deep brain stimulation in traumatic brain injury: a phase 1, randomized feasibility study. *Nat Med*. Dec 2023;29(12):3162-3174.

See Schiff *et al*. Extended Data Figures 3-7 for detailed localizations and histograms for each participant. CL = central lateral thalamic nucleus. DTTm = medial dorsal tegmental tract. MD = mediodorsal thalamic nucleus. VPL = ventral posterior lateral thalamic nucleus. CM = centromedian thalamic nucleus. DBS = deep brain stimulation. P = Participant. L = Left. R = Right.


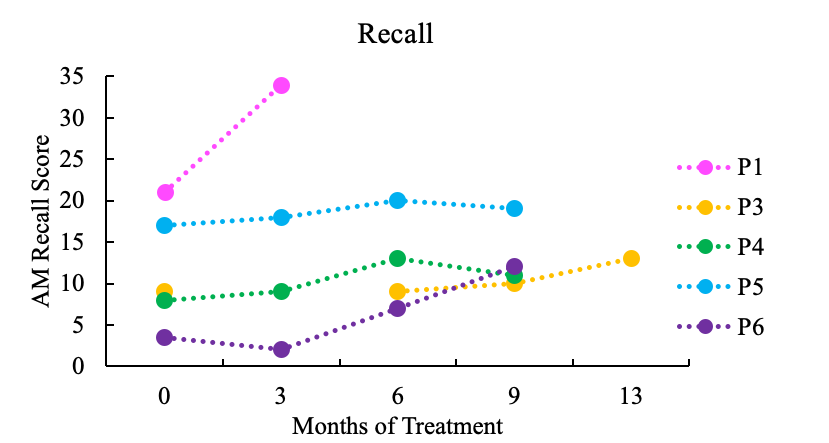


**Supplementary Figure 7. Autobiographical memory recall performance with CL/DTTm DBS treatment over time.** AM recall scores, out of 36 possible total points on the AM Recall Task, are plotted for each participant (P) at various timepoints of treatment. P6’s baseline score is an average of two baseline scores of 3 and 4. Note: the data are underpowered for a statistical analysis of change over time, but are presented here as a graphical illustration of the Correct performance data in Table 3 and Supplementary Table 3. Connecting lines are added to aid in the observation of trends and dashed to indicate that the measures are not continuous.

**Supplementary Table 1 Therapeutic DBS settings**

|  |  |  |  |  |
| --- | --- | --- | --- | --- |
| **Participant** | **Therapeutic Contacts*** | **Amplitude** | **Frequency (Hz)** | **Pulse Width (μs)** |
| **Left** |  |  |  |  |
| P1 | L2-C, L3-C | 3.4 V | 160 | 90 |
| P3 | L2-C, L3-C | 4.0 V | 185 | 60 |
| P4 | L1-A, L2-C, L3-C | 3.5 V | 150 | 60 |
| P5 | L2-C, L3-C | 3.1 – 3.2 V | 150 | 60 |
| P6 | L2-C, L3-C | 3 mA each contact | 150 | 90 |
| **Right** |  |  |  |  |
| P1 | R10-C, R11-C | 3.3 V | 160 | 60 |
| P3 | R10-C, R11-C | 4.0 V | 185 | 80 |
| P4 | R9-A, R10-C | 3.5 V | 150 | 60 |
| P5 | R10-C, R11-C | 3.1 – 3.2 V | 150 | 60 |
| P6 | R9-C, R10-C, R11-C | 3 mA | 150 | 90 |

*A = anode. C = cathode.

Reproduced with permission from Schiff ND, Giacino JT, Butson CR*, et al*. Thalamic deep brain stimulation in traumatic brain injury: a phase 1, randomized feasibility study. *Nat Med*. Dec 2023;29(12):3162-3174.

**Supplementary Table 2 Participant-specific distant and recent time periods in the AM Recall task**

| **Participant** | **Distant Time Period (Pre-injury) / Years Ago** | **Recent Time Period (Post-injury)** |
| --- | --- | --- |
| P1 | High School / 25 | 6mo – 1yr ago |
| P3 | Age 30’s / 30 | 6mo – 1yr ago |
| P4 | High School / 7 | 6mo – 1yr ago |
| P5 | High School / 15 | 6mo – 1yr ago |
| P6 | High School / 15 | 6mo – 1yr ago |

**Supplementary Table 3 Number of trials per response type in the AM Recall task**

| **Participant** | | **Baseline 1** | **Baseline 2** | **3 Months Treatment** | **6 Months Treatment** | **9 Months Treatment** | **13 Months Treatment** | |
| --- | --- | --- | --- | --- | --- | --- | --- | --- |
| P1 |  |  |  |  |  |  |  | |
|  | Timepoint Exceptions |  |  |  |  |  |  | |
|  | DBS on/off during testing | OFF |  | OFF |  |  |  | |
|  | Correct | 21 |  | 34 |  |  |  | |
|  | Omit | 14 |  | 2 |  |  |  | |
|  | Incorrect | 1 |  | 0 |  |  |  | |
| P3 |  |  |  |  |  |  |  | |
|  | Timepoint Exceptions |  |  |  | 5 months* | 8 months* |  | |
|  | DBS on/off during testing | OFF |  |  | ON | ON | ON |  |
|  | Correct | 9 |  |  | 9 | 10 | 13 |  |
|  | Omit | 11 |  |  | 10 | 9 | 9 |  |
|  | Incorrect | 16 |  |  | 17 | 17 | 14 |  |
| P4 |  |  |  |  |  |  |  |  |
|  | Timepoint Exceptions |  |  |  |  |  |  |  |
|  | DBS on/off during testing | OFF |  | ON | ON | OFF |  |  |
|  | Correct | 8 |  | 9 | 13 | 11 |  |  |
|  | Omit | 15 |  | 15 | 19 | 16 |  |  |
|  | Incorrect | 13 |  | 12 | 4 | 9 |  |  |
| P5 |  |  |  |  |  |  |  |  |
|  | Timepoint Exceptions |  |  |  | 7 months* | 10 months* |  |  |
|  | DBS on/off during testing |  | OFF | ON | ON | ON |  |  |
|  | Correct |  | 17 | 18 | 20 | 19 |  |  |
|  | Omit |  | 11 | 14 | 14 | 11 |  |  |
|  | Incorrect |  | 8 | 4 | 2 | 6 |  |  |
| P6 |  |  |  |  |  |  |  |  |
|  | Timepoint Exceptions |  |  |  |  |  |  |  |
|  | DBS on/off during testing | OFF | OFF | OFF | ON | OFF |  |  |
|  | Correct | 3 | 4 | 2 | 7 | 12 |  |  |
|  | Omit | 0 | 17 | 5 | 23 | 20 |  |  |
|  | Incorrect | 33 | 15 | 29 | 6 | 4 |  |  |

Total number of trials per testing session = 36.

Correct refers to an AM recalled from the indicated memory time period within the 15-second time window. Incorrect refers to any other recollection that does not meet the criteria for a successful AM recall, such as a vague memory, semantic information, AM from a wrong memory time period, or a correct AM that appeared in a previous trial. Omit refers to nothing recalled during the 15-second time window.

See Supplementary Figure 7 for a plot of the Correct data.

*Small deviations in the 6-month and 9-month time points for P3 and P5.

**Supplementary Table 4 Percent of distant/pre-injury recalled AMs in the AM Recall task**

| **Participant** | **Baseline** | **Treatment** |
| --- | --- | --- |
| P1 | 23.8% | 47.1% |
| P3 | 33.3% | 46.9% |
| P4 | 63.6% | 58.8% |
| P5 | 52.9% | 63.8% |
| P6 | 100%* | 38.1% |
| Average | 54.7% | 50.9% |
| Paired t-test (*P*) |  | 0.82 |

No significant difference in the frequency of memory time periods (distant/pre-injury or recent/post-injury) of successfully recalled AMs with CL/DTTm DBS treatment in the AM Recall task. Significance was assessed with a two-tailed paired t-test.

*Calculated from recalled AMs from two baseline timepoints.

**Supplementary Table 5 Alertness and valence of recalled AMs in the AM Recall task**

|  | **Alertness** | | |  | **Valence** | | |
| --- | --- | --- | --- | --- | --- | --- | --- |
| **Participant** | **Baseline** | **Average Treatment** | **Percent Change** |  | **Baseline** | **Average Treatment** | **Percent Change** |
| P1 | - | - | - |  | - | - | - |
| P3 | 4.6 | 3.9 | -15.2 |  | 0.9 | 0.4 | -55.6 |
| P4 | 3.3 | 3.2 | -3.0 |  | 0.3 | 0.1 | -66.7 |
| P5 | 3 | 3.3 | 10.0 |  | 0.0 | 0.3 | - |
| P6 | 2.3* | 3.1 | 34.8 |  | 1.7* | 1.3 | -23.5 |
| Paired t-test (*P*) |  |  | 0.83 |  |  |  | 0.34 |

No significant change in the alertness during testing or valence of successfully recalled AMs with CL/DTTm DBS treatment in the AM Recall task. Alertness was measured on a 5-point scale from 1 to 5. Valence was measured on a 6-point scale from -2 to 2. Significance was assessed with a two-tailed paired t-test.

*Average rating of recalled AMs from two baseline timepoints.

**Supplementary Table 6 Vividness of AMs in the Autobiographical Interview task**

| **Participant** | **Baseline** | **Average Treatment** | **Percent Change** |
| --- | --- | --- | --- |
| P3 | 4.3 | 5 | 16.3 |
| P4 | 3.5 | 3.5 | 0.0 |
| P5 | 3.8 | 3.7 | -2.6 |
| P6 | 4.3* | 2.8 | -34.9 |
| Average | 4.0 | 3.8 | -5.3 |
| Paired t-test (*P*) |  |  | 0.33 |

No significant change in the average vividness ratings (“how clearly can you visualize this event on a 1-6 scale?”) of AMs in the Autobiographical Interview with CL/DTTm DBS treatment. Significance was assessed with a one-tailed paired t-test.

*Average score from AMs from two baseline timepoints.
